# Does cardiometabolic risk profile differ among individuals with traumatic and non-traumatic spinal cord injury (SCI): the evidence from the multicenter SCI cohort in Switzerland (SwiSCI)

**DOI:** 10.1038/s41393-024-00996-5

**Published:** 2024-05-15

**Authors:** Peter Francis Raguindin, Oche Adam Itodo, Inge Eriks-Hoogland, Taulant Muka, Mirjam Brach, Gerold Stucki, Jivko Stoyanov, Marija Glisic

**Affiliations:** 1 Swiss Paraplegic Research, Guido A. Zäch Strasse 1, 6207 Nottwil, Switzerland; 2 Institute of Social and Preventive Medicine, University of Bern, Mittelstrasse 43, 3012 Bern, Switzerland; 3 Faculty of Health Sciences and Medicine, University of Lucerne, Alpenquai 4, 6005 Lucerne, Switzerland; 4 Graduate School for Health Sciences, University of Bern, Mittelstrasse 43, 3012 Bern, Switzerland; 5 Swiss Paraplegic Centre, Guido A. Zäch Strasse 1, 6207 Nottwil, Switzerland

**Keywords:** Metabolic syndrome, Epidemiology

## Abstract

**Study design:**

Longitudinal study.

**Objective:**

To explore whether individuals with traumatic spinal cord injury (TSCI) and non-traumatic SCI (NTSCI) experience different trajectories in changes of cardiometabolic disease (CMD) factors during initial rehabilitation stay.

**Setting:**

Multicenter Swiss Spinal Cord Injury Cohort (SwiSCI) study.

**Methods:**

Individuals without history of cardiovascular diseases were included. CMD factors and Framingham risk score (FRS) were compared between TSCI and NTSCI. Linear mixed models’ analysis was employed to explore the trajectory in CMD factors changes over rehabilitation period and a multivariate linear regression analysis was used at discharge from inpatient rehabilitation to explore factors associated with CMD risk profile in TSCI and NTSCI. We performed age and sex-stratified analyses.

**Results:**

We analyzed 530 individuals with SCI (64% with TSCI and 36% NTSCI). The median age was 53 years (IQR:39-64) with 67.9% (n = 363) of the study cohort being male. The median rehabilitation duration was 4.4 months (IQR 2.4-6.4). At admission to rehabilitation, FRS (9.61 vs. 5.89) and prevalence of hypertension (33.16% vs. 13.62%), diabetes (13.68% vs. 4.06%), and obesity (79.05% vs. 66.67%) were higher in NTSCI as compared to TSCI, No difference was observed in cardiometabolic syndrome between the groups (around 40% in both groups). Overall, we observed longitudinal increases in total cholesterol, HDL-C and HDL/total cholesterol ratio, and a decrease in fasting glucose over the rehabilitation period. No differences in longitudinal changes in cardiovascular risk factors were observed between TSCI and NTSCI.

**Conclusions:**

There was no deterioration in cardiometabolic risk factors over rehabilitation period, at discharge from initial rehabilitation stay. Both TSCI and NTSCI experienced high burden of cardiometabolic syndrome components with NTSCI experiencing more disadvantageous risk profile. The effectiveness of therapeutic and lifestyle/behavioral strategies to decrease burden of cardiometabolic disease and its components in early phase should be explored in future studies.

## Introduction

In the past decades, respiratory problems, renal failure and urinary complications were among the leading causes of death in the spinal cord injured (SCI) population [1]. With prolonged life-expectancy and improved acute care, mortality trends in SCI population in developed countries increasingly mimic those of the general population [2], with cardiovascular diseases (CVD) and diabetes being among the leading causes of death in both, traumatic (TSCI) and non-traumatic SCI (NTSCI) [1, 3, 4]. Increased cardiovascular risk post-injury has been driven by autonomic dysfunction, chronic inflammation and prolonged oxidative stress post-injury and has been shown to worsen within weeks post-injury [5]. In the early injury phase, a person participates in specialized rehabilitation program aimed to improve one’s independence in performing activities and to minimize limitations of physical impairments [6]. Factors such as injury severity, secondary health conditions (e.g., urinary tract infections, respiratory complications and pressure ulcers), injury management (e.g., surgical decompression) and medication use (e.g., opioids or steroids’ use) influence patients’ recovery, functioning and metabolic profile [7–10]. Thus, initial rehabilitation stay may be a critical time window to integrate early screening and preventive strategies targeting cardiometabolic risk factors to overcome accelerated functional and metabolic decline following the injury [11].

Only a few studies in the literature have explored changes in the cardiometabolic risk profile during the early phases of TSCI; whereas, there is a clear gap in evidence on cardiometabolic disease (CMD) burden in NTSCI [10, 12–14]. Individuals with traumatic injury may experience different trajectories of CMD risk profile post-injury as compared to those suffering non-traumatic injury due to phenotypic differences between TSCI and NTSCI. For instance, the incidence of NTSCI increases with age, and there is a higher proportion of affected females as compared to TSCI; both age and sex are established CMD risk determinants [15, 16]. Due to the presence of more complete injuries and a higher frequency of complications, patients with TSCI are hospitalized for an average of 3.4 weeks longer than patients with NTSCI, which may further impact cardiometabolic risk profile [17]. Thus, the underrepresentation of NTSCI in research limits the generalizability of findings for the most important determinants of increased CMD risk in SCI and complicates development of effective personalized preventive strategies.

Therefore, our study aims to: (i) determine the prevalence of CMD at admission to initial rehabilitation stay and (ii) explore differences in cardiometabolic risk profile changes over the rehabilitation period comparing individuals within traumatic and non-traumatic injury. We hope that our findings can assist in the identification of individuals with SCI who would benefit the most from preventive approaches to reach the metabolic equilibrium during the early injury phase.

## Methods

### Study design and study cohort

We used data from the inception cohort of the Swiss Spinal Cord Injury (SwiSCI) study [18]. SwiSCI study is a cohort established as a collaboration among four major rehabilitation centers across Switzerland (Swiss Paraplegic Centre, Nottwil; Klinik für Neurorehabilitation und Paraplegiologie-REHAB Basel, Basel; Clinique romande de readaptation, Sion; and Balgrist University Hospital, Balgrist) which serve as regional catchment areas for individuals requiring specialized care post-injury. SwiSCI Inception Cohort prospectively enrolled individuals with SCI who were admitted for inpatient rehabilitation in one of its participating centers in Switzerland. Data were collected in the study centers at four time points following the date of SCI diagnosis: at 28 days (range 16–40 days, T1), 84 days (70–98 days, T2), 168 days (150–186 days, T3), and at discharge (10–0 days before discharge, T4). Our analyses focused on admission to rehabilitation (T1), which represents the study baseline, and rehabilitation discharge (T4). Data are collected by extraction of routine clinical information from the medical records, by clinical assessments, and by paper-and-pencil questionnaires. A comprehensive list of commonly utilized metrics within the collaborating centers was developed, with a focus on prioritizing and standardizing established measures across all four centers. The SwiSCI Inception cohort data model is based on the International Classification of Functioning, Disability and Health (ICF), and the Brief ICF SCI Core Sets in the early post-acute context was used as a reference for the clinical setting. Additionally, whenever applicable and accessible, preference was given to incorporating the “International SCI Basic Data Sets” recommended by the International Spinal Cord Injury Society (ISCOS) (https://www.iscos.org.uk/international-sci-data-sets). Detailed information on the study design and collected data have been reported elsewhere [18, 19].

### Inclusion and exclusion criteria

We enrolled all adults (≥18 years old) from May 2013 to September 2020, who were admitted to any of the four participating rehabilitation centers. Individuals with an SCI attributable to a congenital condition, neurodegenerative disorder, or Guillain–Barré syndrome, or who had a new SCI in the context of palliative care, were excluded from the study. Furthermore, individuals with SCI who had malignant neoplasms or those in palliative/end-of life care were excluded. Finally, we excluded those with previous history of CVD to create a homogenous baseline cardiovascular risk profile of our analysis population. This is also in accordance to how most studies in cardiovascular risk profiling were conducted in the literature.

### Clinical measures and injury classification

The SCI characteristics included SCI lesion etiology (e.g., traumatic vs. non-traumatic, causes of the injury), level and completeness of the injury (motor complete and incomplete), and the pattern of NTSCI injury onset (including acute, sub-acute, prolonged). The level of injury was classified as tetraplegia (at level C2-C7) and paraplegia (level T1-S5), and the completeness of injury into complete motor injury (AIS A and B) and incomplete (AIS C and D) based on the International Standards for Neurological Classification of Spinal Cord Injury (ISNCSCI) [20]. In addition, time since injury and duration of rehabilitation at the SwiSCI rehabilitation center were derived from medical records. Further, demographic characteristics such as age at baseline, sex, information on comorbidities and medication use were obtained from the SwiSCI database and were derived from patient’s medical records.

Venous blood samples were obtained from each participant after an overnight fast. Samples were then sent to respective hospital laboratories for lipid and glucose profiles. Waist circumference (WC) was measured after bowel care. Measurement was taken at the end of a normal exhale, between the lower rib and the top of the hip bone. A flexible tape measure with a precision of 0.5 cm was used. Weight was measured using an electric wheelchair scale. The wheelchair’s weight was subtracted from the total weight of the subject with the wheelchair to determine the subject’s weight expressed in kilograms (kg). Body mass index (BMI) was computed employing the standard formula [weight in kilograms/(height in meters)^2^].

### Outcome measures

We identified individuals with CMD using the criteria provided by the SCI-specific clinical guideline [21]. CMD factors included blood pressure, fasting lipid profile, fasting glucose, and anthropometric measures, that was also used individually for longitudinal modeling. The risk of developing the first cardiovascular event within the next 10 years was assessed using the Framingham risk score (FRS) [22]. The FRS of each study participant was computed at discharge from initial rehabilitation stay using the following variables: (a) age, (b) sex, (c) systolic blood pressure (SBP), (d) total cholesterol (mg/dL), (e) high-density lipoprotein cholesterol (mg/dL), (f) diabetes, and (g) current smoking [22].

### Statistical analyses

We summarized continuous variables using median and interquartile range (IQR) as prescribed by the International Spinal Cord Society (ISCOS) Standards of Data Analysis and Reporting [23]. We log-transformed all non-normally distributed continuous variables. Categorical variables were presented as numbers and percentages. To compare the differences in demographic characteristics, injury characteristics, clinical parameters, lifestyle factors, and comorbidities at baseline between TSCI and NTSCI, we used Wilcoxon signed rank test and chi-square test, as appropriate.

We used a paired t-test to compute the longitudinal changes in cardiometabolic parameters from beginning to end of rehabilitation for individuals with TSCI and NTSCI. We also used a multilevel mixed model using random slope of each individual trajectory by residual maximum likelihood estimation. The longitudinal model was adjusted for age, sex, smoking history, alcohol use, time since injury, prevalent and incident CMD, injury completeness and injury level. Furthermore, we included an interaction term (injury etiology and rehabilitation time) to account for time specific changes in CVD risk factors.

According to the level of injury, we explored the longitudinal changes in cardiovascular risk of the study participants. We similarly used multilevel mixed model using random intercept and individuals as clusters. We used anthropometric measures, blood pressure, fasting lipid profile, and fasting glucose as outcome variable. We used injury etiology (TSCI versus NTSCI) as our predictor variable, with similar model adjustments as previously mentioned.

Finally, we investigated on risk factors for changes in the components of CMD. For this, we performed multivariable linear regression using discharge values (anthropometric measures, blood pressure, fasting lipid profile, and fasting glucose), that were fitted among individuals with TSCI and NTSCI separately. Model adjustments were done as previously mentioned. This was done to explore the longitudinal association between age, sex, injury severity, rehabilitation duration and lifestyle factors and CMD factors.

All statistical analyses were performed using the Stata 16.1 (StataCorp LLC, College Station, TX) for Windows. All computations were done using two-tailed tests, and a p-value of < 0.05 was considered statistically significant.

### Sensitivity analyses

We performed sex-stratified analyses to determine sex-specific associations. We performed age-stratified analysis to determine age-specific associations based on median population age (55 years). To detect selection bias, we also compared the excluded and the included study cohort. For missing data, we tabulated missing exposures and outcomes, and performed list-wise deletion in our regression analyses.

## Results

### Baseline characteristics

**Fig. 1:**
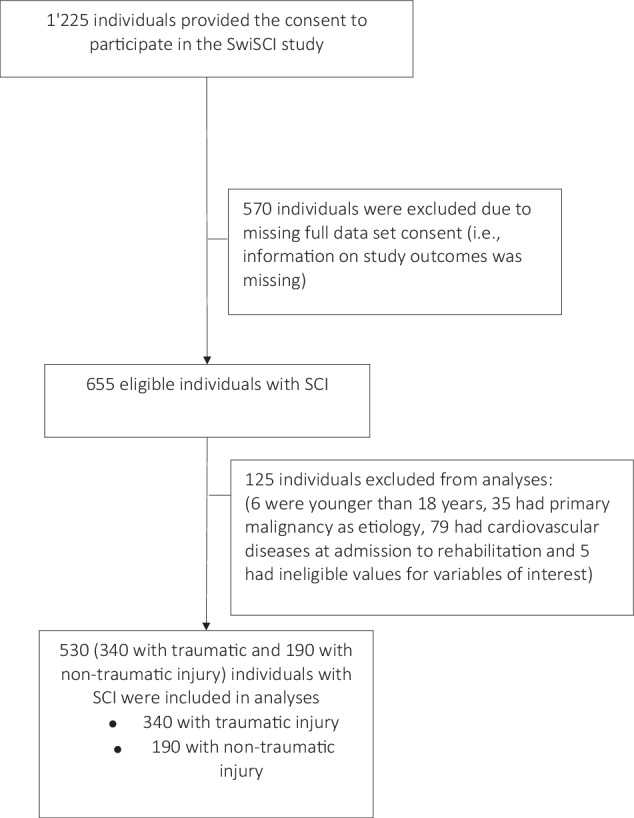
Flow chart of the selection process of the study participants.

The cohort invited 1225 acutely injured individuals from all participating centers. We excluded the following individuals: 570 individuals did not provide full consent for participation, 6 individuals did not meet the age criterion ( < 18 years old), 35 individuals had malignant neoplasms, 79 individuals had prior cardiovascular disease, and 5 individuals had pathologically or physiologically impossible values (erroneous data). Overall, 530 individuals with SCI were included in analyses (Fig. 1).

Among included participants 340 (64%) had traumatic and 190 (36%) non-traumatic injury. The study cohort had a median age of 53 years (IQR 39-64) and the majority were males 363 (67.9%). The median time since injury was 14 days (IQR 9-24) while the median length of rehabilitation stay was 4.4 months (IQR 2.4-6.4). In Table 1 we present the most important clinical characteristics of study participants at admission to rehabilitation stratified by injury etiology (TSCI and NTSCI). In brief, at baseline/admission to rehabilitation, individuals with NTSCI were significantly older (58 years vs. 50 years), had a lower proportion of men (59.5% vs. 72.5%), and had a lower proportion of cervical (23% vs. 36%) and motor complete injuries (10.8% vs. 29.9%). Further, individuals with NTSCI were more likely to have diabetes (13.7% vs. 4.1%), hypertension (33.2% vs. 13.62%), obesity (79.1% vs. 66.7%) and had significantly higher FRS at baseline (9.6% vs. 5.9%). Whereas, individuals with TSCI were more likely to be treated with opioid medications (40.0% vs. 25.8%) and individuals with NTSCI were more likely to be treated with steroids (14.2% vs. 4.7%). In Supplemental Table 1, we present the causes of NTSCI.

**Table 1:** Baseline characteristics of study participants.

Characteristic	All Observations (N = 530)	Traumatic SCI (N = 340, 64%)	Non-traumatic SCI (N = 190, 36%)	P value^a^
Age, years, median (IQR)	53 [39–64]	50 [34–61]	58 [49–68]	<0.01
Males, n (%)	363 (67.9)	250 (72.46)	113 (59.47)	<0.01
Education length, years, median (IQR)	13 (12–16)	13 (12–17)	13 (12–16)	0.39
Education attainment				
• Compulsory	29 (5.4)	20 (5.9)	9 (4.7)	0.45
• Vocational	65 (12.2)	40 (11.7)	25 (13.2)	
• Secondary	137 (25.8)	81 (23.8)	56 (29.5)	
• University	299 (56.4)	199 (58.5)	100 (52.6)	
**SCI Characteristics**				
**Injury level, n (%)**				
• Tetraplegia	166 (31)	123 [36]	43 (23)	<0.01
• Paraplegia	280 [52]	172 [50]	108 [57]	
• Unknown	89 (17)	50 (14)	39 (20)	
Time since injury, days, median (IQR)	14 (9–24)	13 (8–22)	14.50 (9–26.50)	0.02
Length of rehabilitation, months, median (IQR)	4.4 (2.4–6.4)	5.3 (2.9–6.9)	3.3 (1.8–5.0)	<0.01
**Injury completeness, n (%)**				
• Complete	158 (30)	134 [39]	24 (13)	<0.01
• Incomplete	285 [53]	162 [47]	123 [65]	
• Unknown	92 (17)	49 (14)	43 (22)	
**AIS impairment scale,**^b^ **n (%)**				
• A	105 (23.60)	89 (29.97)	16 (10.81)	<0.01
• B	53 (11.91)	45 (15.15)	8 (5.41)	
• C	59 (13.26)	39 (13.13)	20 (13.51)	
• D	225 (50.56)	122 (41.08)	103 (69.59)	
• E	1 (0.22)	1 (0.34)	0 (0.00)	
• Unknown	2 (0.45)	1 (0.34)	1(0.68)	
**Lifestyle factors**				
**Smoking, n (%)**				
• Never	183 [45]	126 [46]	57 [42]	0.43
• Current	224 [55]	146 [54]	78 [58]	
**Alcohol intake reported, n (%)**				
• 1x/month or never	87 (21.27)	46 (16.85)	41 (30.15)	0.02
• 1–3x/month	106 (25.92)	69 (25.27)	37 (27.21)	
• 1–3x/week	128 (31.30)	97 (35.53)	31 (22.79)	
• 4–6x/week	49 (11.98)	35 (12.82)	14 (10.29)	
• Daily	39 (9.54)	26 (9.52)	13 (9.56)	
**Special diet**, n (%)	41 (10.0)	27 (9.9)	14 (10.14)	0.94
**Prevalent CMD**				
Type 2 diabetes mellitus, n (%)^c^	40 (7.48)	14 (4.06)	26 (13.68)	<0.01
Hypertension, n (%)	110 (20.56)	47 (13.62)	63 (33.16)	<0.01
Cardiometabolic syndrome, n (%)^d^	94 (40.87)	59 (39.86)	35 (42.68)	0.68
Overweight/Obesity, n (%)^e^	213 (71.00)	130 (66.67)	83 (79.05)	0.02
10-year Cardiovascular risk (%)^f^	7.11 (2.78–15.05)	5.89 (2.12–14.12)	9.61 (5.08–17.64)	0.01
**Medication use**				
Opioids, n(%)	185 (34.91)	136 (40.00)	49 (25.79)	<0.01
Steroids, n(%)	43 (8.11)	16 (4.71)	27 (14.21)	<0.01

^a^For difference between SCI individuals with traumatic and non-traumatic SCI using the Wilcoxon signed rank test and chi-square test, as appropriate.

^b^International Standards for Neurological Classification of SCI (ISNCSCI).

^c^Diabetes is defined are those diagnosed of type 2 diabetes and ≥7 mmol/L glucose as cut-off.

^d^Cardiometabolic syndrome was defined as simultaneous presence of ≥ 3 of the following factors: Body mass index: ≥ 22 kg/m^2^, fasting triglycerides ≥ 1.7 mmol/L, reduced high-density lipoprotein (“good”) cholesterol (Male ≤ 1.03 mmol/L or in female ≤ 1.29 mmol/L), elevated blood pressure: ≥ 130 mm Hg or use of medication for hypertension, fasting glucose: ≥ 5.6 mmol/L or use of medication for hyperglycemia.

^e^Overweight/Obesity were derived using SCI specific cutoffs (waist circumference ≥ 86.5 cm OR BMI ≥ 22 kg/m^3^).

^f^A 10-year Cardiovascular risk (Framingham risk score) difference was assessed using sign test.

### Longitudinal changes in cardiometabolic risk factors

**Fig. 2:**
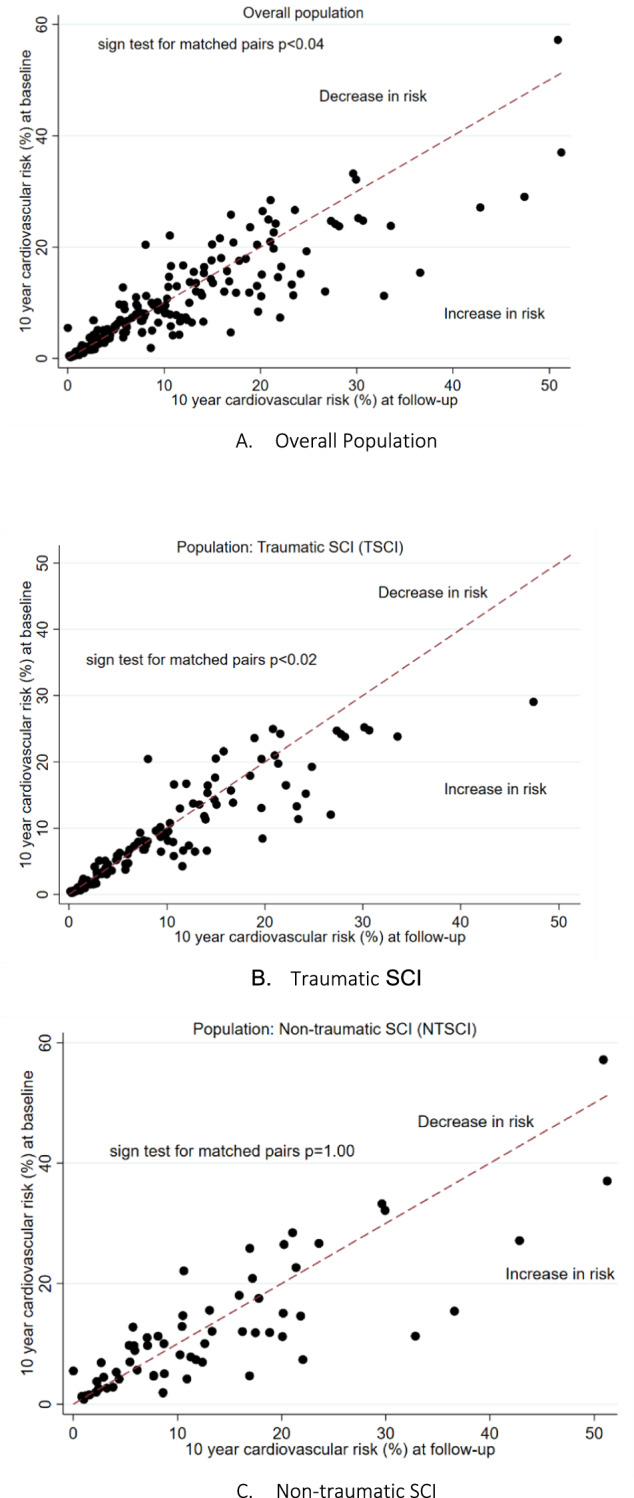
Longitudinal changes in Framingham risk score. **A**–**C** Showing Framingham risk score at baseline and follow-up (10-year risk for first cardiovascular event). The graphs describe the course of each individual represented by a dot via plotting the baseline and follow-up values on x-y-axis. Individuals (dots) on the line mean no change across time has been observed. Individuals above the line represent an increased 10-year risk (Framingham score), whereas those below the line represent a decreased 10-year risk (Framingham score). p value is measured through sign test for matched pairs.

Overall, we observed an increase in total cholesterol, HDL, and HDL-TC ratio between the beginning and end of rehabilitation period [β 0.06 (95%CI 0.03, 0.09) p < 0.01], [β 0.16 (95%CI 0.12, 0.19) p < 0.01] and [β 0.11 (95%CI 0.07, 0.15) p < 0.01], respectively. Glucose concentration decreased over rehabilitation stay [β −0.03 (95%CI −0.06, −0.01) p < 0.01], while no changes in BMI, waist circumference, SBP, DBP, triglycerides, and LDL cholesterol concentration were observed (Table 2). When comparing whether changes in CMD factors differed between individuals with traumatic and non-traumatic injury, in a fully adjusted model, we observed greater increase in mean HDL concentration in NTSCI as compared to TSCI [β 0.08 (95%CI 0.00, 0.16) p < 0.05]. We did not observe any changes among other risk factors (Table 3). Sex and age stratified analysis was in line with overall findings (Supplemental Tables 2 and 3). The Fig. 2 depicts individual trajectories in FRS score comparing the beginning and end of rehabilitation, in the overall cohort, and individuals with TSCI and NTSCI. Overall, we observed decreasing FRS over the rehabilitation stay in the overall study cohort and among those with traumatic injury, whereas for NTSCI no significant change in FRS was observed.

**Table 2:** Longitudinal changes in CMD risk factors considering all available study participants.

	Beginning of Rehabilitation^a^	End of Rehabilitation^a^	p-value^b^	Fully corrected^c^	p-value
Body mass index	24.1 (21.3–27.3)	24.3 (21.3–27.5)	0.71	0.00 [−0.01, 0.01]	0.89
Waist circumference	89.8 [80–99]	90.1 [81–99]	0.48	−0.01 [−0.02, 0.01]	0.37
Systolic blood pressure	119 (108–130)	120 (110–130)	0.08	0.01 [−0.00, 0.03]	0.12
Diastolic blood pressure	70 [60–78]	70 [63–80]	0.06	0.01 [−0.01, 0.03]	0.27
Total cholesterol	4.5 (3.9–5.2)	4.8 (4.1–5.5)	**<0.001**	**0.06 [0.03, 0.09]**	**<0.01**
Triglycerides	1.5 (1.1–2.0)	1.4 (1.0–2.0)	0.15	−0.05 [−0.11, 0.00)	0.05
HDL cholesterol	1.1 (0.8–1.3)	1.2 (1.0–1.4)	**<0.001**	**0.16 [0.12, 0.19]**	**<0.01**
LDL cholesterol	2.8 (2.3–3.4)	2.8 (2.2–3.5)	0.64	0.02 [−0.02, 0.05]	0.32
HDL/Total cholesterol ratio	0.22 (0.18–0.29)	0.25 (0.20–0.32)	**<0.001**	**0.11 [0.07, 0.15]**	**<0.01**
Fasting glucose	5.0 (4.6–5.7)	5.0 (4.6–5.6)	**0.02**	**−0.03 [−0.06, −0.01]**	**0.01**

^a^Values expressed as median and interquartile range.

^b^Comparison done by paired t-test using log-transformed values. P values < 0.05 in bold are considered statistically significant.

^c^Adjustments using age, sex, smoking history, alcohol use, time since injury, injury etiology (TSCI vs NTSCI), diabetes mellitus, injury etiology, injury completeness and injury level. We also included interaction term of exposure (injury etiology and length of rehabilitation).

**Table 3:** Longitudinal changes in CMD risk factors comparing NTSCI vs TSCI (a reference group).

Parameter	Unadjusted Difference,^a^ β (95% CI)	p value	Adjusted Difference,^b^ β (95% CI)	p value
Body mass index	0.03 [−0.04,0.10]	0.48	0.01 [−0.08,0.10]	0.80
Waist circumference	0.03 [−0.03,0.09]	0.31	−0.04 [−0.11,0.03]	0.30
Systolic blood pressure	**0.03 [0.01,0.05]**	**0.01**	−0.00 [−0.03,0.02]	0.51
Diastolic blood pressure	0.01 [−0.02,0.03]	0.66	−0.02 [−0.06,0.01]	0.11
Total Cholesterol	0.00 [−0.04,0.05]	0.92	−0.01 [−0.07,0.04]	0.58
Triglycerides	−0.02 [−0.12,0.07]	0.66	−0.11 [−0.20,0.03]	0.07
HDL Cholesterol	**0.09 [0.03,0.16]**	**0.01**	**0.08 [0.00,0.16]**	**<0.05**^c^
LDL Cholesterol	−0.01 [−0.08,0.05]	0.73	0.02 [−0.07,0.07]	0.96
HDL/Total cholesterol ratio	0.02 [−0.12,0.16]	0.77	0.02 [−0.18,0.18]	0.83
Fasting glucose	0.02 [−0.02,0.06]	0.34	−0.01 [−0.06,0.04]	0.58

*HDL* high-density lipoprotein, *LDL* low-density lipoprotein.

Values were log-transformed for the linear mixed model. P values < 0.05 (**in bold**) are considered statistically significant.

^a^Crude (univariable) linear mixed model.

^b^Adjusted (multivariable) linear mixed model. Adjusted for age, sex, smoking status, alcohol use, and medications (statins and hypertension at baseline and follow-up), and diabetes, duration of injury, injury level and completeness, and interaction of length of rehabilitation and exposure (etiology).

^c^p-value is at 0.049.

### Cardiometabolic Risk Profile Prior to Discharge from Initial Rehabilitation

We explored the association between one’s clinical characteristics and cardiometabolic risk factors through multivariable linear regression (adjusting for age, sex, smoking history, alcohol use, time since injury, diabetes, injury etiology, injury completeness and injury level). For TSCI, we found a positive association between age and SBP, DBP, LDL, and WC [β 0.22 (95%CI 0.12, 0.31) p < 0.001], [β 0.17 (95%CI 0.10, 0.24) p < 0.001], [β 0.01 (95%CI 0.00, 0.01) p < 0.05], [β 0.36 (95%CI 0.27, 0.44) p < 0.001] respectively (Table 4). SBP, DBP and triglycerides were lower in females [β −4.34 (95%CI −7.94, −0.75) p < 0.05], [β −3.05 (95%CI −5.82, −0.28) p < 0.05], and [β −0.35 (95%CI −0.67, −0.02) p < 0.05] respectively; while HDL and HDL-total cholesterol ratio were higher in females vs. males [β 0.35 (95%CI 0.21, 0.48) p < 0.001] and [β 0.05 (95%CI 0.01, 0.08) p < 0.05], respectively. Total cholesterol was higher among individuals with paraplegia in comparison to tetraplegia [β 0.38 (95%CI 0.09, 0.68) p < 0.01]. Total cholesterol and triglycerides were higher among smokers [β 0.45 (95%CI 0.05, 0.84) p < 0.05] and [β 0.64 (95%CI 0.24, 1.04) p < 0.01], respectively.

**Table 4:** Association between personal and clinical characteristics and cardiometabolic risk factors at discharge.

Traumatic injury									
	Systolic blood pressure β (95% CI)	Diastolic blood pressure β (95% CI)	Fasting glucose β (95% CI)	Total cholesterol β (95% CI)	Triglycerides β (95% CI)	HDL β (95% CI)	LDL β (95% CI)	HDL Total cholesterol ratio β (95% CI)	Waist circumference β (95% CI)
Age	0.22 (0.12, 0.31)***	0.17 (0.10, 0.24)***	0.01 (−0.00, 0.02)	0.01 (−0.00, 0.01)	−0.00 (−0.01, 0.00)	0.00(−0.00, 0.00)	0.01 (0.00, 0.01)*	−0.00 (−0.00,0.00)	0.36 (0.27,0.44)***
Females vs males (ref)	−4.34 (−7.94,−0.75)*	−3.05 (−5.82, 0.28)*	0.19 (−0.22, 0.59)	0.27 (−0.05, 0.59)	−0.35 (−0.67, −0.02)*	0.35 (0.21, 0.48)***	0.09(−0.17, 0.35)	0.05 (0.01,0.08)*	−3.81 (−7.42,−0.20)
Individuals with paraplegia vs tetraplegia (ref)	0.41 (−3.13, 3.94)	0.73 (−1.98, 3.44)	−0.05 (−0.42, 0.33)	0.38 (0.09, 0.68)**	0.24 (−0.06, 0.55)	0.06 (−0.06, 0.19)	0.22 (−0.02, 0.46)	−0.00 (−0.03, 0.03)	0.28 (−3.30, 3.86)
Incomplete vs complete injury (ref)	−0.51 (−3.98, 2.97)	1.64 (−1.02, 4.31)	−0.17 (−0.54, 0.19)	0.09 (−0.20, 0.38)	0.05 (−0.25, 0.35)	0.09 (−0.04,0.22)	−0.06 (−0.30, 0.18)	0.01 (−0.03,0.04)	−1.29 (−4.82, 2.24)
Injury duration	0.01 (−0.06, 0.08)	0.04 (−0.02, 0.09)	−0.01 (−0.01, 0.00)	0.00 (−0.00, 0.01)	0.00 (−0.00, 0.01)	−0.00 (−0.00, 0.00)	0.00 (−0.00, 0.01)	−0.00 (−0.00,0.00)	0.00 (−0.07,0.08)
Rehabilitation length	0.36 (−1.82, 2.55)	1.08 (−0.62, 2.78)	−0.18 (−0.40, 0.04)	0.05 (−0.12, 0.22)	0.06 (−0.12, 0.24)	−0.01 (−0.08,0.07)	0.02 (−0.12,0.16)	−0.01 (−0.03,0.01)	0.14 (−2.08,2.36)
Smoker vs. non-smokers (ref)	−2.33 (−7.00, 2.34)	−0.99 (−4.55, 2.57)	−0.05 (−0.58, 0.48)	0.45 (0.05, 0.84)*	0.64 (0.24, 1.04)**	−0.07 (−0.24, 0.11)	0.24 (−0.08,0.56)	−0.04 (−0.08,0.01)	−1.00 (−5.45,3.45)
Alcohol drinkers vs non-alcohol drinkers (ref)	2.90 (−1.81, 7.61)	0.71 (−2.92,4.34)	0.14 (−0.38, 0.66)	−0.34 (−0.76, 0.07)	−0.29 (−0.72, 0.13)	0.13 (−0.05, 0.31)	−0.31(−0.65, 0.03)	0.02 (−0.03, 0.07)	−3.10 (−7.83,1.62)
**Non-traumatic injury**									
	**Systolic blood pressure β (95% CI)**	**Diastolic blood pressure β (95% CI)**	**Fasting glucose β (95% CI)**	**Total cholesterol β (95% CI)**	**Triglycerides β (95% CI)**	**HDL β (95% CI)**	**LDL β (95% CI)**	**HDL Total cholesterol ratio β (95% CI)**	**Waist circumference β (95% CI)**
Age	0.19 (0.03, 0.35)*	0.20 (−0.21, 0.61)	0.01 (−0.00,0.02)	0.01(−0.01,0.02)	0.00(−0.01,0.02)	0.00(−0.00,0.01)	0.00(−0.01,0.01)	0.00(−0.00,0.00)	0.18(−0.00,0.36)
Females vs males (ref)	−1.64 (−6.30, 3.02)	7.81 (−4.01, 19.65)	−0.14 (−0.57,0.28)	0.41(−0.01,0.84)	−0.16(−0.51,0.19)	0.25(0.06,0.44)*	0.13(−0.25,0.52)	0.02(−0.04,0.08)	−3.90(8.94,1.14)
Individuals with paraplegia vs tetraplegia (ref)	1.93 (−3.78,7.65)	−15.38 (−29.91, −0.86)*	−0.42 (−0.91,0.07)	0.04(−0.47,0.56)	0.20(−0.22,0.62)	−0.13(−0.36,0.09)	−0.04(−0.52,0.43)	−0.03(−0.10,0.04)	0.59(−5.62,6.80)
Incomplete vs complete injury (ref)	3.53 (−3.66, 10.73)	9.81 (−8.67, 28.28)	−0.12 (−0.73,0.49)	0.23(−0.40,0.87)	0.07(−0.46,0.59)	0.25(−0.04,0.53)	0.05(−0.54,0.64)	0.01(−0.07,0.10)	−6.65(−14.28,0.98)
Toxic and metabolic vs genetic disorder (ref)	−1.35 (−6.08, 3.39)	3.98 (−8.17,16.14)	0.12 (−0.30,0.54)	−0.49(−0.91,−0.06)*	−0.01(−0.36,0.35)	−0.12(−0.32,0.07)	−0.40(−0.80,−0.01)*	0.02(−0.04,0.07)	−3.30(−8.52,1.92)
Injury duration	0.00 (−0.00,0.01)	−0.00 (−0.02, 0.01)	−0.00(−0.00,0.00)	−0.00(−0.00,0.00)	0.00(−0.00,0.00)	−0.00(−0.00,0.00)	−0.00(−0.00,0.00)	−0.00(−0.00,0.00)	0.00(−0.00,0.01)
Non-acute vs. acute injury (ref)	1.39 (−3.27, 6.04)	−8.49 (−20.28,3.31)	0.15(−0.26,0.56)	0.11(−0.31,0.53)	0.07(−0.27,0.41)	−0.01(−0.21,0.17)	0.12(−0.26,0.50)	0.00(−0.05,0.06)	−0.28(−5.35,4.79)
Rehabilitation length	0.09 (−0.08, 0.26)	−0.09 (−0.53,0.35)	−0.00(−0.01,0.01)	−0.00(−0.02,0.01)	0.01(−0.00,0.02)	−0.00(−0.01(0.00)	−0.00(−0.02,0.01)	−0.00(−0.00,0.00)	0.12(−0.05,0.29)
Smoker vs non-smokers (ref)	1.66 (−4.74, 8.07)	−0.82 (−5.32,3.68)	0.16(−0.27,0.60)	0.18(−0.35,0.71)	0.44(0.02,0.85)*	0.14(−0.13,0.41)	0.07(−0.49,0.63)	0.05(−0.02,0.13)	−5.41(−12.44,1.63)
Alcohol drinkers vs non-alcohol drinkers (ref)	1.63 (−3.86,7.12)	2.36 (−11.62,16.35)	0.03(−0.44,0.50)	−0.08(−0.57,0.41)	0.04(−0.35,0.44)	0.07(−0.15,0.29)	−0.17(−0.61,0.28)	0.03(−0.04,0.09)	−5.00(−10.81,0.81)

*HDL* high-density lipoprotein, *LDL* low-density lipoprotein.

Adjustments using age, sex, smoking history, alcohol use, time since injury, injury etiology (TSCI vs NTSCI), diabetes mellitus, injury etiology, injury completeness and injury level. We also included interaction term of exposure (injury etiology and length of rehabilitation).

*P value < 0.05 ** P < 0.01 *** P < 0.001.

*For dichotomized variables, reference groups were labeled as “ref”. Otherwise, the covariate was fitted in the model as continuous variables (i.e., age, injury duration, and rehabilitation length.

In NTSCI, increasing age was associated with higher SBP [β 0.19 (95%CI 0.03, 0.35) p < 0.05] and females compared to males had higher HDL [β 0.25 (95%CI 0.06, 0.44) p < 0.05]. Furthermore, DBP was lower among those with paraplegia in comparison to those with tetraplegia [β−15.38 (95%CI −29.91, −0.86) p < 0.05]. Total cholesterol and LDL were lower among individuals with toxic and metabolic NTSCI injury etiology [β −0.49 (95%CI −0.91, −0.06) p < 0.05] and [β −0.40 (95%CI −0.80, −0.01) p < 0.05] respectively (Table 4). Finally, triglycerides were higher among smokers [β 0.44 (95%CI 0.02, 0.85) p < 0.05]. We did not find any further associations for the remaining cardiometabolic risk factors.

### Sensitivity analyses

The percentage of missing data for the dependent variables included in the analysis was generally lower than 50% (Supplemental Table 4). The excluded individuals with SCI were older compared to individuals who were included in the analysis [62 years IQR (43–72) vs 53 years IQR (41–64)]. We did not find any significant differences in sex, education level, injury level nor completeness among the two comparison groups, Supplemental Table 5.

## Discussion

This study provides the first comparison of cardiometabolic disease burden among individuals with TSCI and NTSCI admitted to first inpatient rehabilitation stay. At admission, we observed significantly higher proportion of individuals with tetraplegia and motor complete injury among those with TSCI as compared to NTSCI. Individuals with NTSCI were older, comprised higher proportion of females and higher burden of cardiometabolic risk factors (obesity, diabetes, hypertension). They also had significantly higher FRS, which indicates the risk of developing the first CVD events within the next 10 years. Following an average of 4.4 months in rehabilitation, we observed a borderline improvement in lipid profile (higher HDL-C and HDL-C/TC ratio) and glucose in the overall study cohort and no significant differences in changes in these factors between NTSCI and TSCI. Factors such as age, male sex, injury level, and smoking were associated with poorer risk profile at discharge.

### Clinical implications of our findings and outlook

First, previous studies focusing on CMD burden during initial rehabilitation stay were conducted among individuals with traumatic injury. Our findings among individuals with TSCI are comparable with those reported in the literature [12, 24], however, our findings among individuals with NTSCI are to be confirmed in other populations.

Second, higher burden of CMD at admission among those with NTSCI is not a surprise and may be driven by older age at the time of injury, as well as the higher proportion of females (e.g., after menopause females observe undesirable changes in body composition and CMD risk profile). We used the FRS to calculate the 10-year risk of first CVD event. Individuals with NTSCI had significantly higher scores at admission. In Fig. 2, we provide individual trajectories in FRS (comparing admission and discharge) for the overall cohort and among those with TSCI and NTSCI. Over the follow-up period (on average 5.3 months in TSCI and 4.4 months in NTSCI) we observed a decrease in FRS in TSCI and no change in score among NTSCI. The FRS may underestimate the future CVD risk in individuals with SCI. Thus, the true risk may be even higher than reported in the current study. Moreover, the follow-up period may not be sufficient to detect more extensive changes in FRS. Yet, regardless of FRS, considering high burden of hypertension, diabetes, and overweight and obesity among those with NTSCI future studies are needed to determine on whether routine CVD screening should be implemented during initial rehabilitation stay.

Third, nearly every individual sustaining SCI receives multiple types of medications that may alter or modify blood glucose, lipids, and blood pressure. These drugs manage a range of problems associated with neurotrauma to the spinal cord, secondary health conditions (pain, muscle spasm, respiratory or unitary tract infections) and multimorbidity. Most prescribed medications include skeletal muscle relaxants, analgesic-narcotics or tricyclic antidepressants [25]. Simultaneous use of these medications may have a detrimental effect on neurological recovery and functioning as well as increase the risk of complications such as respiratory depression, fractures, hypothalamic–pituitary–gonadal (HPG) axis dysregulation and lead to adverse metabolic changes [25]. Among these medications, opioids, although not recommended as a first-line therapy for pain due to questionable benefit-to-risk ratio, are ubiquitously administered for pain management in humans sustaining an acute SCI during a therapeutic window of opportunity for neuroprotection and repair [25, 26]. We observed that high proportions of individuals with TSCI (40%) and NTSCI (25%) were treated with opioid medications. Another study from Canada reported that in the year following discharge from inpatient rehabilitation, 60% of individuals with NTSCI had opioid medications prescribed [27]. Older age, being female, diagnosis of osteoporosis, prior exposure to prescription opioids, higher morbidity score, and lower functional status were the main predictors of higher opioid use [27]. Thus, future studies should explore the patterns of opioid prescriptions and the clinical significance of opioid medications on modifying metabolic changes, rehabilitation outcomes and functioning post-injury. Finally, considering the high burden of CMD during initial rehabilitation stay, it is worthwhile to explore the patterns of treatment and prophylactic use of CMD medications (e.g., antihypertensives, statins, etc.) as well as lifestyle and behavioral interventions.

### Study strengths and limitations

To our knowledge, this is the first study to describe cardiometabolic risk factors in individuals with NTSCI and the first to compare them to individuals with traumatic injury. Major strengths of our analysis include robust statistical estimates by applying linear mixed models for repeated measures analysis and a relatively large sample size. However, this study has some limitations. First, it is possible that because we used the SCI-specific cut-offs for defining obesity (BMI > 22 kg/m^2^ [21, 28] and WC ≥ 86.5 cm [29, 30]) and metabolic disease/syndrome, some individuals may be misclassified and the proportion of individuals with obesity may be overestimated. In particular, we cannot ascertain when the SCI specific cut-off should be used along the course of SCI or how early it should be used to define overweight/obesity. Second, we used the FRS that may be a suboptimal risk estimator in the SCI population. However, despite FRS underestimating the overall risk, it can still distinguish between those who are at high vs. low CVD risk in the SCI population [31]. Third, because causes for NTSCI are heterogeneous, including tumor-related, congenital/developmental, infectious, inflammatory and ischemic causes, as well as several others, interpretation of changes in CMD factors may be challenging [32]. Despite all efforts to harmonize data collection within the four rehabilitation centers, we cannot exclude the possibility of variation in clinical assessments across the involved centers. Fifth, females, the older adults, and individuals with lower functional independence were less likely to participate in the SwiSCI study [18]. Therefore, it is possible that initially, individuals with an impaired CMD risk profile were less likely to be included in the SwiSCI. Although we excluded individuals with a history of CVD from the current study, the generalizability of our results could be limited when considering individuals with a poorer CMD risk profile.

## Conclusion

In conclusion, we report a high prevalence of cardiometabolic disease and its components at admission to first inpatient rehabilitation, especially in individuals with NTSCI. We did not observe differences in early changes in CMD components among TSCI and NTSCI. Moreover, there is a systematic lack of evidence on cardiometabolic diseases in NTSCI and our results should be replicated in another study whereas the potential of early preventable strategies (e.g., CMD prophylactic medication use, or lifestyle modifications) to improve metabolic health among those with NTSCI should be explored.

### Supplementary information

Online supplement

## Data Availability

The dataset generated and/or analyzed during the current study are available from the SwiSCI Study Center on reasonable request.
