# Supplementary material for: Does cardiometabolic risk profile differ among individuals with traumatic and non-traumatic spinal cord injury (SCI): the evidence from the multicenter SCI cohort in Switzerland (SwiSCI)
Source: Spinal Cord. 2024 May 15;62(7):387–95. doi: 10.1038/s41393-024-00996-5 (PMC11230898; doi:10.1038/s41393-024-00996-5)
Supplement: Supplementary file 1 — Online supplement [file 41393_2024_996_MOESM1_ESM.docx]

**Does cardiometabolic risk profile differ among individuals with traumatic and non-traumatic spinal cord injury (SCI): the evidence from the multicenter SCI cohort in Switzerland (SwiSCI)**

Peter Francis Raguindin^1,2,3^, Oche Adam Itodo^1,2,4^, Inge Eriks Hoogland^1,3,5^, Taulant Muka^2^, Mirjam Brach^1^, Gerold Stucki^1^, Jivko Stoyanov^1^, Marija Glisic^1,2^

^1^ Swiss Paraplegic Research, Guido A. Zäch Strasse 1, 6207 Nottwil, Switzerland

^2^Institute of Social and Preventive Medicine, University of Bern, Mittelstrasse 43, 3012 Bern, Switzerland

^3^Faculty of Health Sciences and Medicine, University of Lucerne, Alpenquai 4, 6005 Lucerne, Switzerland

^4^Graduate School for Health Sciences, University of Bern, Mittelstrasse 43, 3012 Bern, Switzerland

^5^Swiss Paraplegic Centre, Guido A. Zäch Strasse 1, 6207 Nottwil, Switzerland

Contents

[Supplemental Table 1. Causes of Non-traumatic Spinal cord Injury 2](#_Toc120442791)

[Supplemental Table 2. Sex-stratified analysis on the cardiometabolic risk according to injury etiology across time (longitudinal analysis)^1^ 3](#_Toc120442792)

[Supplemental Table 3. Age-stratified analysis on the cardiometabolic risk according to injury etiology across time (longitudinal analysis)^1^ 4](#_Toc120442793)

[Supplemental Table 4 . Summary of missing values at beginning and end of rehabilitation 5](#_Toc120442794)

[Supplemental Table 5 . Analysis of selection bias of included and excluded participants 6](#_Toc120442795)

| **Medical condition** | **Frequency n= 190 (%)** |
| --- | --- |
| **Spinal cord compression due to combination of multiple factors** | 20 (11) |
| **Neoplasm** | 18 (9) |
| **Bacterial infection** | 12(6) |
| **Spinal stenosis** | 23(12) |
| **Demyelination** | 4(2) |
| **Vascular malformations** | 7(4) |
| **Vertebral column degenerative disorders** | 30(16) |
| **Inflammatory and Auto-immune Diseases** | 7(4) |
| **Metabolic disorders** | 1(1) |
| **Vascular disorders (unknown)** | 5(3) |
| **Vascular disorders (Ischaemia)** | 43(23) |
| **Vascular disorders (Haemorrhage)** | 10(5) |
| **Infection (unknown)** | 1(1) |
| **Miscellaneous (Unknown)** | 8(4) |
| **Radiation related** | 1(1) |

# Supplemental Table 1. Causes of Non-traumatic Spinal cord Injury

# Supplemental Table 2. Sex-stratified analysis on the cardiometabolic risk according to injury etiology across time (longitudinal analysis)^1^

|  | TSCI | NTSCI | p |
| --- | --- | --- | --- |
| Body mass index |  |  |  |
| - Overall population | Ref | 0.0115 [-0.0756,0.0987] | 0.795 |
| - Males | Ref | 0.0340 [-0.0290, 0.0970] | 0.290 |
| - Females | Ref | 0.0910 [-0.0134, 0.1953] | 0.087 |
| Waist circumference |  |  |  |
| - Overall population | Ref | -0.0373 [-0.107,0.0326] | 0.296 |
| - Males | Ref | 0.0245 [-0.0119, 0.0610] | 0.187 |
| - Females | Ref | -0.0179 [-0.0715, 0.0357] | 0.512 |
| Systolic blood pressure |  |  |  |
| - Overall population | Ref | -0.00820 [-0.0325,0.0161] | 0.508 |
| - Males | Ref | -0.0217 [-0.0549, 0.0115] | 0.200 |
| - Females | Ref | 0.0071 [-0.0323, 0.0465] | 0.724 |
| Diastolic blood pressure |  |  |  |
| - Overall population | Ref | -0.0247 [-0.0552,0.00594] | 0.114 |
| - Males | Ref | -0.0331 [-0.0745, 0.0083] | 0.117 |
| - Females | Ref | 0.0015 [-0.0510, 0.0540] | 0.955 |
| Total cholesterol |  |  |  |
| - Overall population | Ref | -0.0148 [-0.0672,0.0377] | 0.581 |
| - Males | Ref | -0.0230 [-0.0912, 0.0452] | 0.508 |
| - Females | Ref | 0.0089 [-0.0909, 0.1088] | 0.861 |
| Triglycerides |  |  |  |
| - Overall population | Ref | -0.105 [-0.197,0.0333] | 0.072 |
| - Males | Ref | -0.0958 [-0.2380, 0.0464] | 0.187 |
| - Females | Ref | -0.0890 [-0.2709, 0.0928] | 0.337 |
| Low density lipoprotein |  |  |  |
| - Overall population | Ref | 0.0201 [-0.0746,0.0725] | 0.958 |
| - Males | Ref | -0.0083 [-0.1071, 0.0905] | 0.869 |
| - Females | Ref | 0.0402 [-0.0994, 0.1797] | 0.573 |
| High-density lipoprotein |  |  |  |
| - Overall population | Ref | **0.0787* [0.000440,0.157]** | **0.049** |
| - Males | Ref | 0.0885 [-0.0137, 0.1906] | 0.090 |
| - Females | Ref | 0.0575 [-0.0772, 0.1923] | 0.402 |
| HDL LDL ratio |  |  |  |
| - Overall population | Ref | -0.0550 [-0.282,0.172] | 0.636 |
| - Males | Ref | -0.3023 [-0.6087, 0.0041] | 0.053 |
| - Females | Ref | -0.1441 [-0.5650, 0.2768] | 0.502 |
| HDL Total cholesterol ratio |  |  |  |
| - Overall population | Ref | 0.0199 [-0.177,0.183] | 0.826 |
| - Males | Ref | -0.1598 [-0.3898, 0.0702] | 0.173 |
| - Females | Ref | -0.0631 [-0.3996, 0.2734] | 0.713 |
| Fasting blood glucose |  |  |  |
| - Overall population | Ref | -0.0137 [-0.0626,0.0352] | 0.583 |
| - Males | Ref | -0.0305 [-0.0993, 0.0384] | 0.385 |
| - Females | Ref | -0.0306 [-0.1063, 0.0451] | 0.428 |

^1^Based on fully adjusted model which includes age, sex, smoking status, alcohol use, medications (statins and hypertension at baseline and follow-up), duration of injury, baseline and prevalent T2DM, and injury level and completeness. Values were log-transformed before regression analysis.

# Supplemental Table 3. Age-stratified analysis on the cardiometabolic risk according to injury etiology across time (longitudinal analysis)^1^

|  | TSCI | NTSCI | p |
| --- | --- | --- | --- |
| Body mass index |  |  |  |
| - Overall population | Ref | 0.0115 [-0.0756,0.0987] | 0.795 |
| - Adults (</=55) | Ref | 0.0615 [-0.0713, 0.1943] | 0.364 |
| - Elderly (>55) | Ref | -0.0510 [-0.1773, 0.0754] | 0.429 |
| Waist circumference |  |  |  |
| - Overall population | Ref | -0.0373 [-0.107,0.0326] | 0.296 |
| - Adults (</=55) | Ref | -0.0023 [-0.1076, 0.1029] | 0.965 |
| - Elderly (>55) | Ref | -0.0356 [-0.1324, 0.0613] | 0.472 |
| Systolic blood pressure |  |  |  |
| - Overall population | Ref | -0.00820 [-0.0325,0.0161] | 0.508 |
| - Adults (</=55) | Ref | -0.0045 [-0.0372, 0.0282] | 0.787 |
| - Elderly (>55) | Ref | -0.0016 [-0.0394, 0.0361] | 0.932 |
| Diastolic blood pressure |  |  |  |
| - Overall population | Ref | -0.0247 [-0.0552,0.00594] | 0.114 |
| - Adults (</=55) | Ref | -0.0133 [-0.0585, 0.0320] | 0.566 |
| - Elderly (>55) | Ref | -0.0225 [-0.0633, 0.0183] | 0.280 |
| Total cholesterol |  |  |  |
| - Overall population | Ref | -0.0148 [-0.0672,0.0377] | 0.581 |
| - Adults (</=55) | Ref | -0.0735 [-0.1527, 0.0056] | 0.069 |
| - Elderly (>55) | Ref | 0.0248 [-0.0477, 0.0974] | 0.502 |
| Triglycerides |  |  |  |
| - Overall population | Ref | -0.105 [-0.197,0.0333] | 0.072 |
| - Adults (</=55) | Ref | -0.1713 [-0.3583, 0.0157] | 0.073 |
| - Elderly (>55) | Ref | -0.0116 [-0.1543, 0.1311] | 0.873 |
| Low density lipoprotein |  |  |  |
| - Overall population | Ref | 0.0201 [-0.0746,0.0725] | 0.958 |
| - Adults (</=55) | Ref | -0.0839 [-0.1957, 0.0280] | 0.142 |
| - Elderly (>55) | Ref | 0.0558 [-0.0490, 0.1605] | 0.297 |
| High-density lipoprotein |  |  |  |
| - Overall population | Ref | **0.0787* [0.000440,0.157]** | **0.049** |
| - Adults (</=55) | Ref | 0.0994 [-0.0205, 0.2192] | 0.104 |
| - Elderly (>55) | Ref | 0.0896 [-0.0235, 0.2027] | 0.121 |
| HDL LDL ratio |  |  |  |
| - Overall population | Ref | -0.0550 [-0.282,0.172] | 0.636 |
| - Adults (</=55) | Ref | 0.1030 [-0.2339, 0.4400] | 0.549 |
| - Elderly (>55) | Ref | -0.0793 [-0.4001, 0.2414] | 0.628 |
| HDL Total cholesterol ratio |  |  |  |
| - Overall population | Ref | 0.0199 [-0.177,0.183] | 0.826 |
| - Adults (</=55) | Ref | 0.1197 [-0.1476, 0.3869] | 0.380 |
| - Elderly (>55) | Ref | 0.0001 [-0.2474, 0.2476] | 0.999 |
| Fasting blood glucose |  |  |  |
| - Overall population | Ref | -0.0137 [-0.0626,0.0352] | 0.583 |
| - Adults (</=55) | Ref | -0.0452 [-0.1256, 0.0351] | 0.270 |
| - Elderly (>55) | Ref | 0.0118 [-0.0483, 0.0720] | 0.699 |

^1^Based on fully adjusted model which includes age, sex, smoking status, alcohol use, medications (statins and hypertension at baseline and follow-up), duration of injury, baseline and prevalent T2DM, and injury level and completeness. Values were log-transformed before regression analysis

# Supplemental Table 4 . Summary of missing values at beginning and end of rehabilitation

| Missing values n, (%) | | | |
| --- | --- | --- | --- |
| **Beginning of rehabilitation** | | | |
| **Variables** | **All observations (530)** | **Traumatic SCI**  **(N=340, 64%)** | **Non-traumatic SCI**  **(N=190, 36%)** |
| Total cholesterol | 205 (39) | 126 (37) | 79 (42) |
| Triglycerides | 212 (40) | 128 (38) | 84 (44) |
| HDL | 236 (44.5) | 144 (42) | 92 (48) |
| LDL | 259 (49) | 163 (48) | 96 (51) |
| Fasting blood glucose | 176 (33) | 104 (31) | 72 (38) |
| Systolic blood pressure | 87 (16) | 46 (14) | 41 (22) |
| Diastolic blood pressure | 87 (16) | 46 (14) | 41 (22) |
| Waist circumference | 212 (40) | 134 (39) | 78 (41) |
| BMI | 133 (25) | 79 (23) | 54 (28) |
| HDL-LDL ratio | 259 (48.9) | 152 (45) | 107 (56) |
| Total cholesterol HDL ratio | 237 (44.7) | 145 (43) | 92 (48) |
| **End of rehabilitation** | | | |
| Total cholesterol | 157 (29.6) | 105 (31) | 52 (27) |
| Triglycerides | 161 (30) | 106 (31) | 55 (29) |
| HDL | 160 (30) | 106 (31) | 54 (28) |
| LDL | 168 (31.7) | 111 (33) | 57 (30) |
| Fasting blood glucose | 164 (30.9) | 102 (30) | 62 (33) |
| Systolic blood pressure | 37 (7) | 27 (8) | 10 (5) |
| Diastolic blood pressure | 37 (7) | 27 (8) | 10 (5) |
| Waist circumference | 96 (18) | 75 (22) | 21 (11) |
| BMI | 126 (23.8) | 82 (24) | 44 (23) |
| HDL-LDL ratio | 168 (31.7) | 107 (31) | 61 (32) |
| Total cholesterol HDL ratio | 160 (30) | 104 (31) | 56 (29) |

| Supplemental Table 5 . Analysis of selection bias of included and excluded participants | | | | |
| --- | --- | --- | --- | --- |
| **Characteristic** | **All Observations^1^**  **(N=1093)** | **Included**  **(N=530, 48%)** | **Excluded**  **(N=563, 52%)** | **P value^1^** |
| Age, years | 57 (42-69) | 53 (41-64) | 62 (43-72) | <0.001 |
| Males, n (%) | 726 (66) | 359 (68) | 367 (65) | 0.372 |
| Education, years | 13.8 (3.3) | 13.8 (3.3) | 13.4 (2.4) | 0.509 |
| **SCI Characteristics** | | | | |
| **Injury level** | | | | |
| Tetraplegia, n (%) | 349 (32) | 164 (31) | 185 (33) | 0.370 |
| Paraplegia, n (%) | 548 (50) | 277 (52) | 271 (48) |  |
| ^2^Other, n (%) | 196 (17) | 89 (17) | 107 (19) |  |
| **Injury completeness** | | | | |
| Complete | 329 (30) | 155 (29) | 174 (31) | 0.801 |
| Incomplete | 573 (52) | 283 (53) | 290 (52) |  |
| Unknown | 191 (17) | 92 (17) | 99 (17) |  |
| ^1^for difference between included and excluded SCI individuals with using the Student’s t-test, Wilcoxon signed rank test and chi-square test, as appropriate | | | | |
